# Penicillin Binding Protein Substitutions Cooccur with Fluoroquinolone Resistance in Epidemic Lineages of Multidrug-Resistant Clostridioides difficile

**DOI:** 10.1128/mbio.00243-23

**Published:** 2023-04-05

**Authors:** Kate E. Dingle, Jane Freeman, Xavier Didelot, T. Phuong Quan, David W. Eyre, Jeremy Swann, William D. Spittal, Emma V. Clark, Keith A. Jolley, A. Sarah Walker, Mark H. Wilcox, Derrick W. Crook

**Affiliations:** a Nuffield Department of Clinical Medicine, John Radcliffe Hospital, Oxford University, Oxford, United Kingdom; b National Institute for Health Research (NIHR) Oxford Biomedical Research Centre, John Radcliffe Hospital, Oxford, United Kingdom; c Department of Microbiology, Leeds Teaching Hospitals Trust, Leeds, United Kingdom; d Healthcare Associated Infections Research Group, The Leeds Institute of Medical Research, University of Leeds, Leeds, United Kingdom; e School of Life Sciences and Department of Statistics, University of Warwick, Coventry, United Kingdom; f Big Data Institute, Nuffield Department of Population Health, Oxford University of Oxford, Oxford, United Kingdom; g Department of Biology, University of Oxford, Oxford, United Kingdom; Yale School of Public Health

**Keywords:** AMR mechanism, antimicrobial resistance, *Clostridioides difficile*, PBPs, penicillin binding proteins, cell wall transpeptidase, cephalosporin MIC, cephalosporin resistance

## Abstract

Clostridioides difficile remains a key cause of healthcare-associated infection, with multidrug-resistant (MDR) lineages causing high-mortality (≥20%) outbreaks. Cephalosporin treatment is a long-established risk factor, and antimicrobial stewardship is a key control. A mechanism underlying raised cephalosporin MICs has not been identified in C. difficile, but among other species, this is often acquired via amino acid substitutions in cell wall transpeptidases (penicillin binding proteins [PBPs]). Here, we investigated five C. difficile transpeptidases (PBP1 to PBP5) for recent substitutions, associated cephalosporin MICs, and co-occurrence with fluoroquinolone resistance. Previously published genome assemblies (*n* = 7,096) were obtained, representing 16 geographically widespread lineages, including healthcare-associated ST1(027). Recent amino acid substitutions were found within PBP1 (*n* = 50) and PBP3 (*n* = 48), ranging from 1 to 10 substitutions per genome. β-Lactam MICs were measured for closely related pairs of wild-type and PBP-substituted isolates separated by 20 to 273 single nucleotide polymorphisms (SNPs). Recombination-corrected phylogenies were constructed to date substitution acquisition. Key substitutions such as PBP3 V497L and PBP1 T674I/N/V emerged independently across multiple lineages. They were associated with extremely high cephalosporin MICs; 1 to 4 doubling dilutions >wild-type, up to 1,506 μg/mL. Substitution patterns varied by lineage and clade, showed geographic structure, and occurred post-1990, coincident with the *gyrA* and/or *gyrB* substitutions conferring fluoroquinolone resistance. In conclusion, recent PBP1 and PBP3 substitutions are associated with raised cephalosporin MICs in C. difficile. Their co-occurrence with fluoroquinolone resistance hinders attempts to understand the relative importance of these drugs in the dissemination of epidemic lineages. Further controlled studies of cephalosporin and fluoroquinolone stewardship are needed to determine their relative effectiveness in outbreak control.

## INTRODUCTION

Clostridioides difficile is among the leading causes of health care-associated infection, with symptoms ranging from diarrhea to potentially fatal pseudomembranous colitis (1). Over the last 30 years, unrestricted antimicrobial use has selected multidrug-resistant (MDR) C. difficile lineages, which can be identified by multilocus sequence type (ST) and/or PCR ribotype (2–9). Uncontrolled prescribing of antimicrobials such as fluoroquinolones and cephalosporins, which are associated with a high risk of C. difficile infection (CDI), creates conditions under which MDR lineages can cause persistent, high-mortality (≥20%) outbreaks (9–18). Such health care-associated transmission may be geographically widespread, as in the “hypervirulent” ST1 ribotype 027 [ST1(027)] lineage FQ-R1 (19), and/or prolonged, as in ST17(018), predominating in Japanese and Italian health care settings since the 1990s (17, 20). Cases associated with the rapid transmission of MDR lineages are typically superimposed on a background of sporadic, unlinked cases caused by diverse C. difficile strains, which lack acquired antimicrobial resistance (AMR) (21, 22).

Antimicrobial stewardship is an extremely effective means of preventing or resolving CDI outbreaks in health care settings (23–29). This approach contributed to the marked decline in fluoroquinolone-resistant lineages in the United Kingdom a decade ago, with resistant ST1(027), ST3(001), ST42(106), and ST37(017) falling from 67% to ~3% of cases (30, 31). Fluoroquinolone resistance can be predicted from whole-genome sequences by characteristic single nucleotide polymorphisms (SNPs) in the chromosomal *gyrA* and/or *gyrB* genes (30, 32). Equivalent analysis for cephalosporins is lacking because the genetic mechanism(s) influencing cephalosporin susceptibility in C. difficile is unknown. Wild-type cephalosporin MICs, defined as the phenotype conferred by genes in their ancestral, nonmutated form, as found in the natural C. difficile population, are already moderate to high for this species, up to and greater than 256 μg/mL (33–36). This has led to the widely accepted concept that C. difficile cephalosporin MICs are intrinsically high (10). For this reason, and the practical difficulties of determining MICs approaching the limit of drug solubility, cephalosporin MICs are rarely measured for C. difficile.

Studies aiming to understand the mechanism of C. difficile cephalosporin resistance have focused on the endogenous C. difficile class D β-lactamase, but findings have been inconclusive (37, 38). In many bacterial species, reduced susceptibility to cephalosporins and other β-lactams is conferred by amino acid substitutions in penicillin binding proteins (PBPs). These are enzymes catalyzing cell wall peptidoglycan biosynthesis that are classified according to molecular weight (high [HMW] or low [LMW]) and enzymatic activity (transpeptidase or carboxypeptidase) (39). β-Lactam antibiotics target PBPs by acting as inhibitory substrate analogues (39), binding covalently to the active site serine (40) in the first of three conserved motifs, SXXK, (S/Y)XN, and (K/H)(S/T)G (41). Exposure to β-lactams selects substitutions which reduce the affinity of the drug for the PBP, increasing the MIC (42, 43). However, the nature and frequency of PBP substitutions among clinically important C. difficile lineages, and their impact on cephalosporin MICs, have not been investigated systematically. Here, our aims were to identify and characterize PBP substitutions in C. difficile, determine the extent to which they cooccur with fluoroquinolone resistance in epidemic C. difficile lineages, and date this phylogenetically. Additionally, we aimed to assess the phenotype of both PBP-substituted strains and closely related wild-type controls in terms of their cephalosporin MICs.

## RESULTS

The study was designed as follows. A globally distributed collection of published C. difficile genomes was assembled (*n* = 7,094) representing 16 genetic lineages, 14 commonly associated with CDI in health care settings and 2 carried asymptomatically (nontoxigenic). The occurrence of recent, within-lineage PBP substitutions was investigated and compared to the occurrence of fluoroquinolone resistance. Cephalosporin (and other β-lactam) MICs were measured for representative strains containing PBP substitutions and closely related “wild-type” ancestors. Finally, the timing and sequence of PBP substitution and fluoroquinolone resistance acquisition events were investigated phylogenetically.

### Lineages studied.

The 7,094 genomes represented lineages ST1(ribotypes 027/198/176/181) (*n* = 1,918), ST17(018) (*n* = 279), ST42(106) (*n* = 563), ST3(001) (*n* = 411), ST37(017) (*n* = 424) and its recent descendant ST81(369) (*n* = 39), ST63(053) (*n* = 37), ST54(012) (*n* = 148), ST8(002) (*n* = 593) and its recent descendant ST183 (*n* = 14), ST11(078) (*n* = 628), ST2(014/020) (*n* = 790), ST6(005) (*n* = 404), ST10(015) (*n* = 263), ST7(026) (*n* = 190), ST56(058) (*n* = 16), and two prevalent nontoxigenic genotypes, ST26(039) (*n* = 175) and ST15(010) (*n* = 202). Four genetically distinct C. difficile clades, 1, 2, 4, and 5, were represented (44). Genomes, accession numbers, AMR predictions from genotype and references are listed per lineage (see Data Set S1, sheets 1 to 18, in the supplemental material).

10.1128/mbio.00243-23.2 DATA SET S1 Metadata, resistance determinants, PBP substitutions, and SRA accession numbers for all the genomes studied. Data are organized by lineage. Numbers below the column headings *gyrA*, *gyrB*, *ermB*, *rpoB*, PBP1, and PBP3 indicate the allele sequence identified; sequences are available at https://pubmlst.org/bigsdb?db=pubmlst_cdifficile_seqdef&page=downloadAlleles. The columns headed “aa” indicate amino acid substitutions differing from the wild-type sequence. References are supplied on the final tab. Download Data Set S1, XLSX file, 1.6 MB. Copyright © 2023 Dingle et al. 2023 Dingle et al. https://creativecommons.org/licenses/by/4.0/ This content is distributed under the terms of the Creative Commons Attribution 4.0 International license.

### C. difficile PBPs.

To date, nine PBPs have been described within the C. difficile genome (Table 1), five of which are transpeptidases (PBP1 to PBP5 [PBP1–5]) (45). HMW PBP1 and PBP3 are essential for growth *in vitro* (46), while LMW PBP2 and PBP4 are not essential for growth but are required for sporulation (46). Only LMW PBP5 has been described as variably present (45). PBP1 to PBP4 were present in all genomes studied, while PBP5 occurred in lineages ST3(001), ST11(078), and ST37(017)/ST81(369). No additional PBPs were identified using known C. difficile PBP sequences in low-stringency BLAST searches.

**TABLE 1:** PBPs and β-lactamases of C. difficile reference genomes^*a*^^,^^*i*^

Strain R20291 locus NC_013316.1	Strain CD630 locus NC_009089.1 (former locus)	Alternate designation	PBP classification	Size (aa)	blastp-predicted function/family	blastp E value^*h*^
0712^*b*^	RS04495 (07810)^*e*^	PBP1^*g*^	HMW class A	897	Bifunctional transglycosylase/transpeptidase pbp_1A_fam	2.14e–166
0985^*b*^	RS06420 (11480)^*e*^	PBP3^*g*^	HMW class B	992	Transpeptidase Pbp2_mrdA for cell elongation	1.53e–127
1067^*c*^	RS06830 (12290)	PBP2^*g*^	LMW class B	554	FtsI/Pbp2	2.85e–101
					Transpeptidase Pbp2_mrdA for cell elongation	1.81e-93
2544^*c*^	RS14215 (26560)^*e*^	PBP4^*g*^ *spoVD*	LMW class B	659	spoVD_pbp transpeptidase	0e+00
					FtsI/Pbp2	3.23e–165
-	-	PBP5^*f*^^,^^*g*^ strain M68	LMW class B	696	FtsI/Pbp2	1.00e–110
					Pbp2_mrdA transpeptidase	2.67e–104
1131^*c*^	RS07160 (12910)^*e*^	*dacF*	LMW class C	387	d-Alanyl-d-alanine carboxypeptidase	1.68e–116
2048^*c*^	RS11615 (21410)		LMW class C	397	d-Alanyl-d-alanine carboxypeptidase	2.31e–88
0441^*d*^	RS03150 (05150)		LMW class C	414	d-Alanyl-d-alanine carboxypeptidase	8.55e–94
2390^*d*^	RS13415 (24980)	*dacF1*	LMW class C	429	d-Alanyl-d-alanine carboxypeptidase	7.59e–97
3056^*c*^	RS17015 (31960)		PBP or β-lactamase?	340	blastp: CubicO group peptidase, β-lactamase class C (R20291 “put. PBP,” CD630 “serine hydrolase”)	3.06e–40
1318^*d*^	RS08060 (14690)	*cwp20*	PBP or β-lactamase?	1013	blastp: β-lactamase, cell wall binding protein repeats (R20291 “put. PBP,” “cell surface protein”)	2.40e–55
2283	RS12870 (23930)			338	Transglycosylase domain-containing protein	1.37e–74
0399	RS02840 (04580)	blaCDD, CDD1/2	β-Lactamase	312	YbaI class D β-lactamase (37)	5.94e–51

a Gray shading indicates proteins containing transpeptidase domains. MDR reference strains contain the following PBP substitutions relative to the wild-type of the identical genotype: strain R20291, ST1(027) UK 2006 (96) contains PBP3 V497L; strain CD630, ST54(012) Switzerland 1982 (99) contains PBP1 T674I and PBP3 N537K; strain M68, ST37(017) Ireland 2006 (99) contains PBP3 Y721C.

b Essential for growth *in vitro* (46).

c Not essential for growth *in vitro* but required for sporulation (46).

d Not essential for growth *in vitro* (46).

e Existence shown experimentally by mass spectrometry (104, 105).

f PBP5 absent in R20291 and CD630 but present in M68 (NC_017175.1) chromosomal locus RS02615, coordinates 501965 to 504052.

g PBP1–5 (45).

h blastp E values for R20291 sequences: the closer the value is to zero, the more significant the match.

i Dashes indicate that PBP5 is absent from strain R20291 and strain CD630.

### Recent PBP substitutions.

PBP gene sequences were compared within each lineage to identify recent SNPs. These were absent or rare in LMW PBP2, PBP4, and PBP5. In contrast, multiple SNPs occurred in HMW PBP1 and PBP3, almost all of which were nonsynonymous. The resultant amino acid substitutions affected a total of 48/993 (4.8%) positions in PBP3 and 50/855 to 925 (5.8 to 5.4%) positions in PBP1. Substitution data are shown per isolate (Data Set S1). The variable PBP1 size reflected differing numbers of repeats of the 14 amino-acid sequence, TPPDNGGNNGGGST, which occurred between 1 and 6 times near the C terminus of the protein (Table S1).

10.1128/mbio.00243-23.3 TABLE S1 PBP1 C-terminal repeat sequences, exemplified by the PBP1 alleles shown. The variable PBP1 length reflected variable 42-nt repeats which occurred between one and six times, near the 3’ end of the gene. Each repeat encoded 14 amino acids, with the sequence TPPDNGGNNGGGST. The numbers of repeats varied within ST and did not follow patterns of MDR. When blastp searches were performed using PBP1 alleles containing different numbers of repeats (1 to 4 and 6), matches with 100% identity were identified for each, suggesting that the observed repeat is not specific to the methods used here. The details of the top three blastp matches for each number of repeats are shown. Download Table S1, DOCX file, 0.02 MB. Copyright © 2023 Dingle et al. 2023 Dingle et al. https://creativecommons.org/licenses/by/4.0/ This content is distributed under the terms of the Creative Commons Attribution 4.0 International license.

**FIG 1:**
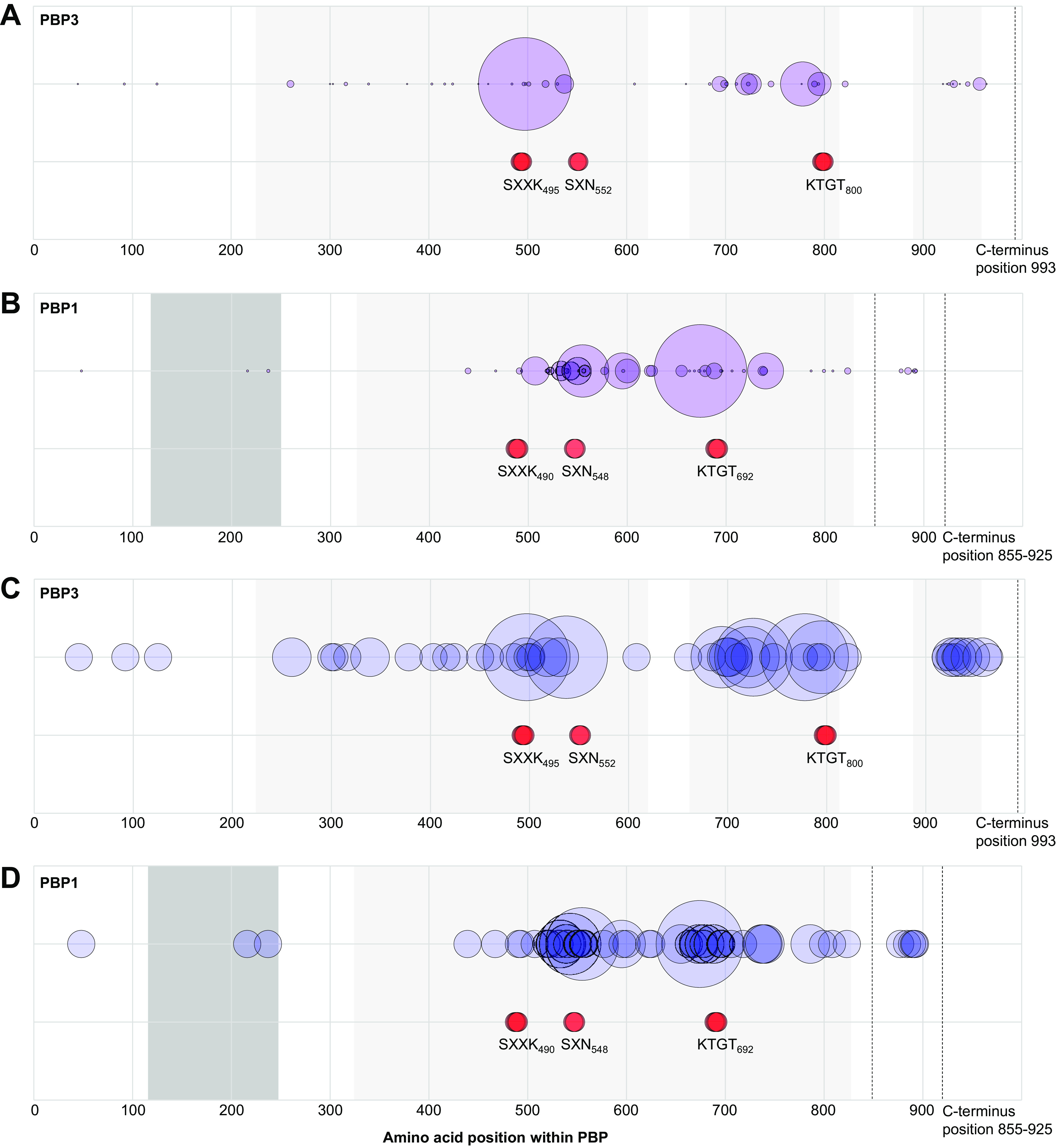
Positions and relative frequency of amino acid substitutions within PBP3 (993 amino acids) and PBP1 (855 to 925 amino acids). (A) Substitutions within PBP3 (*n* = 48) are represented by purple circles, plotted according to locations within the PBP (*x* axis). Circles are scaled according to substitution frequency within the entire data set. Light gray shading indicates the position of conserved transpeptidase domains identified by BLASTP. Red circles indicate transpeptidase catalytic motifs. (B) Same as described for panel A but for PBP1 (*n* substitutions = 50) and dark gray indicates the N-terminal glycosyl transferase domain. (C) Same as described for panel A except that relative sizes of blue circles indicate the number of lineages in which each substitution was identified. (D) Same as described for panel B, with blue circles again indicating the number of lineages in which each substitution was identified. Raw data, including the identity of each substitution, are shown in Table 2.

The frequency of each PBP amino acid substitution was recorded per lineage (Table 2). This identified the most common substitutions within and among lineages. In PBP3, V497L was most frequent, occurring in 2,897 genomes and 10 lineages, followed by A778V in 664 genomes and 10 lineages. In PBP1, substitution T674I/N/V was most frequent, occurring in 1,379 genomes of 10 lineages, followed by A555T in 442 genomes of 7 lineages. These data were plotted to visualize the relative positions and frequencies of substitutions within PBP1 and PBP3 (Fig. 1). Virtually all substitutions occurred within the conserved transpeptidase domains, flanking the active site motifs.

**TABLE 2:**
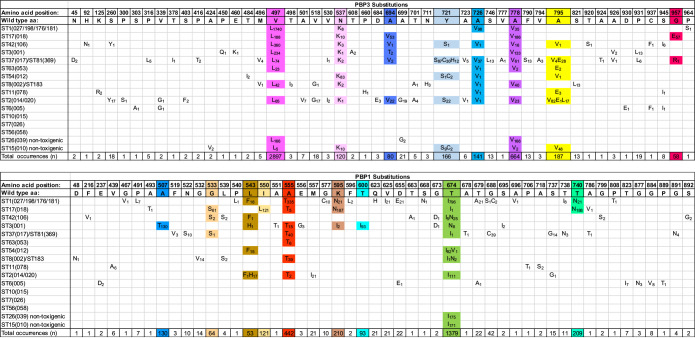
PBP3 and PBP1 substitutions identified within each of the 16 lineages studied^*a*^

a Upper panel, PBP3; lower panel, PBP1 (with lineages in the left-hand column for both panels). The amino acid positions across the top of each panel indicate positions within PBP3 or PBP1, together with the wild-type amino acid. Amino acid substitutions are indicated within each panel; their within-lineage frequency is shown as a subscript. The overall frequency of each substitution within the entire data set is indicated in the bottom row. Colored boxes indicate the substitutions occurring more than 50 times within the data set.

### Association between PBP substitutions and fluoroquinolone resistance.

**FIG 2:**
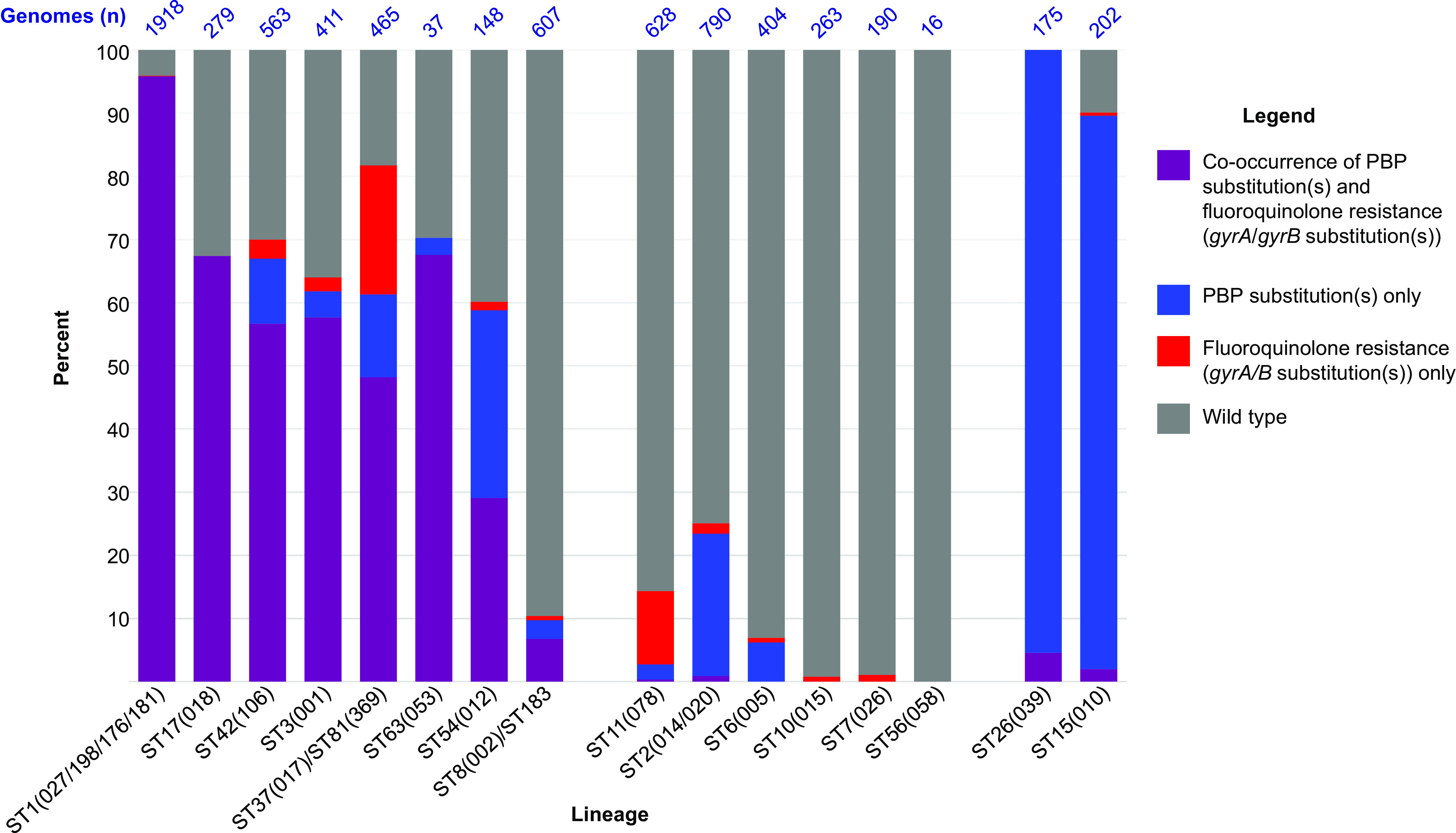
Occurrence of PBP substitutions and fluoroquinolone resistance in the 16 lineages studied.

In addition to PBP substitutions, the presence of fluoroquinolone resistance was investigated in all lineages (Fig. 2; Data Set S1). The occurrence of PBP substitutions was significantly associated with fluoroquinolone resistance in 8 of the 14 clinically important lineages: ST1(027/198/176/181), ST17(018), ST42(106), ST3(001), ST37(017)/ST81(369), ST63(053), ST54(012), and ST8(002)/ST183 (Fig. 2, *P* < 0.001), suggesting the possibility of almost simultaneous acquisition. There was no evidence of association for the remaining six clinically important lineages, ST11(078) (*P* = 1.00), ST2(014/020) (*P* = 0.281), and ST6(005) (*P* = 1.00), with ST10(015), ST7(026), and ST56(058) containing no isolates with PBP substitutions (*P* = 0.865 for the group overall). Similarly, there was no evidence of association for the two nontoxigenic lineages, ST15(010) (*P* = 0.426) and ST26(039) (no isolates with fluoroquinolone resistance substitutions) (*P* = 0.531 for the group overall).

Among the 14 clinically important lineages studied, PBP substitutions occurred less often in the absence of fluoroquinolone resistance (Fig. 2; Data Set S1). Genomes in this category reached between 6 and 30% of the total examined only in lineages ST42(106), ST37(017), ST54(012), ST2(014/020), and ST6(005). Only two toxigenic lineages, ST37(017) and ST11(078), were notable for fluoroquinolone resistance in the absence of PBP substitutions, at 20 and 12% of the total genomes examined, respectively (Fig. 2; Data Set S1). PBP substitutions occurred without fluoroquinolone resistance in over 90% of nontoxigenic lineage genomes ST26(039) and ST15(010) (Fig. 2; Data Set S1).

### Association between PBP substitutions and β-lactam MICs.

**FIG 3:**
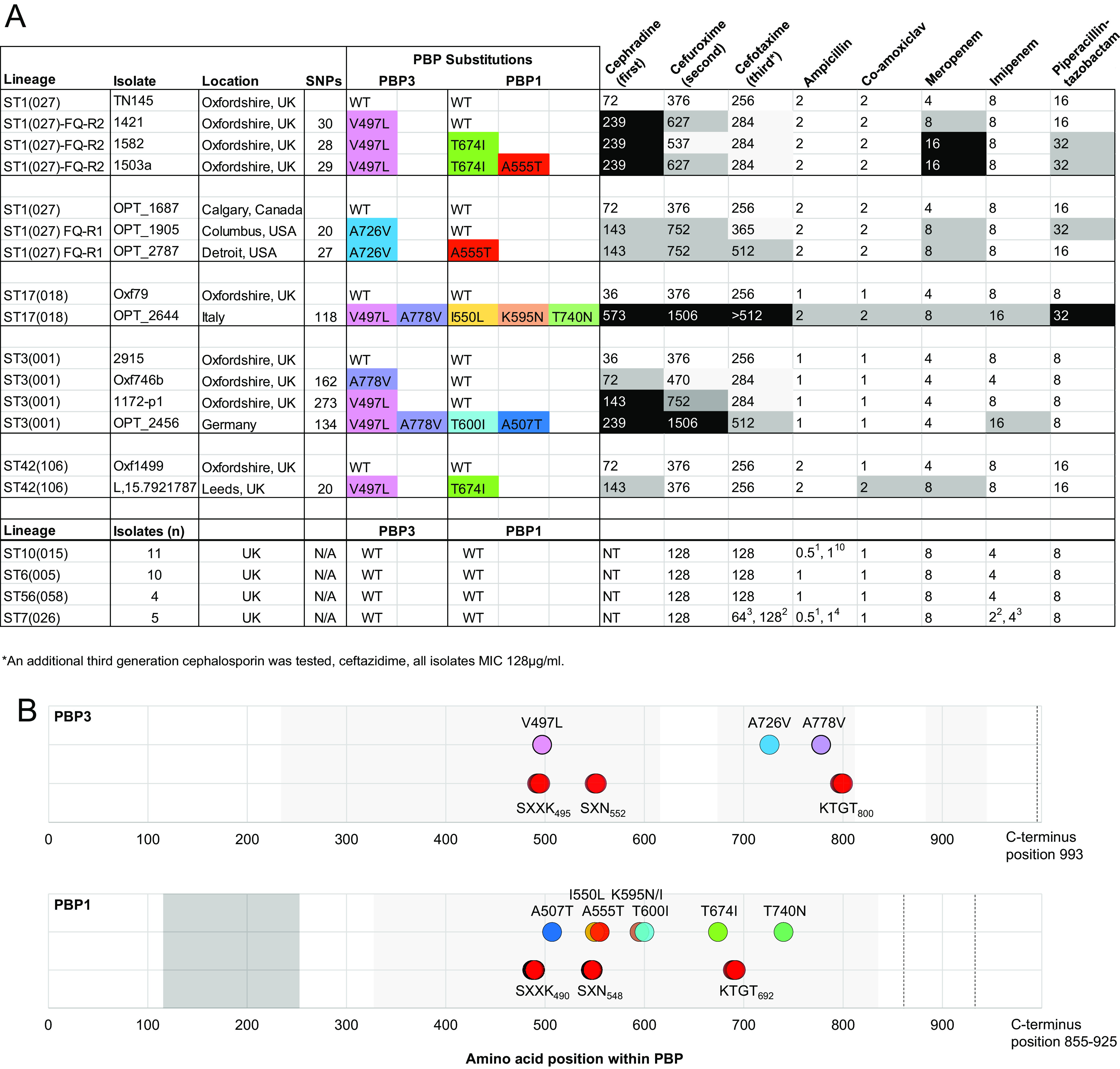
β-Lactam MICs of wild-type and PBP-substituted C. difficile isolates. (A) Upper panel, MICs for the β-lactams shown, measured for isolates belonging to the toxigenic MDR lineages ST1(027) (FQ-R2 and FQ-R1), ST17(018), ST3(001), and ST42(106), with one row per isolate tested. The column headed “SNPs” refers to the number of single nucleotide polymorphisms between each isolate and its wild-type (WT) within-lineage comparator. Identical PBP substitutions found in different isolates are highlighted by identical colors. The intensity of gray shading indicates the fold increase in MIC relative to the MIC of the wild type. Lower panel, MICs measured for the toxigenic non-MDR lineages ST10(015), ST6(005), ST56(058), and ST7(026), with one row per lineage. The number of isolates tested per lineage, if greater than 1, is indicated as a superscript. NT, not tested. (B) Positions of the PBP substitutions (colored as shown in panel A) contained in the isolates that underwent phenotyping (A), relative to the conserved transpeptidase domains (gray) and the active site motifs (red circles).

β-Lactam MICs were measured for isolates representing eight of the lineages studied. Four lineages, ST1(027) (FQ-R1 and FQ-R2 [19]), ST3(001), ST42(106), and ST17(018), were chosen due to their clinical importance and high proportion of genomes containing both PBP substitutions and fluoroquinolone resistance (Fig. 2). Specific PBP substitution combinations were chosen for phenotyping on the basis of high frequency within available collections (Data Set S1). Individual isolate choices were based on the low numbers of SNP differences (Fig. 3A) between wild-type and PBP-substituted genomes. The remaining four lineages we phenotyped were ST10(015), ST6(005), ST56(058), and ST7(026). These were chosen because while their prevalence varied (Fig. 2), they all lacked PBP substitutions and therefore represented additional wild-type controls. Certain PBP substitutions/combinations were identified only in genomes we obtained from publicly available sequence databases (for example, the Southeast Asian clades ST81(369) and ST183). For this reason, their phenotype could not be assessed.

Among the isolates phenotyped from lineages ST1(027) (FQ-R1 and FQ-R2 [19]), ST3(001), ST42(106), and ST17(018), 10 different individual PBP substitutions were represented, three in PBP3 and seven in PBP1 (Fig. 3A and B). These included the four substitutions identified most frequently in the study as a whole (Fig. 1A and B; Data Set S1; Fig. 3B). For ST1(027) FQ-R2, MICs were determined for 3 different PBP substitution combinations (1, 2, or 3 substitutions) versus the wild-type; for ST1(027) FQ-R1, MICs were determined for 2 PBP combinations (1 or 2 substitutions) versus the wild-type; for ST17(018), MICs were determined for 1 PBP combination (containing 5 substitutions) versus the wild-type; for ST3(001), MICs were determined for 3 PBP combinations (containing 1, 1, or 4 substitutions) versus the wild-type; and finally, for ST42(106), MICs were determined for 1 PBP substitution combination (containing 2 substitutions) versus the wild-type (Fig. 3A). For the lineages lacking PBP substitutions, the numbers of isolates phenotyped were as follows: ST10(015), *n* = 11; ST6(005), *n* = 10; ST56(058), *n* = 4; and ST7(026), *n* = 5 (Fig. 3A).

The number of PBP substitutions per isolate was significantly correlated with MIC for cephalosporins (for cephradine, *r* = 0.88, *P* < 0.001; cefuroxime, *r* = 0.76, *P* = 0.001; cefotaxime, *r* = 0.78, *P* < 0.001) and slightly less strongly for carbapenems (for meropenem, *r* = 0.65, *P* = 0.009; imipenem, *r* = 0.50, *P* = 0.059). In comparison, there was no significant correlation for penicillins (amoxicillin: *r* = 0.17, *P* = 0.55; co-amoxiclav: *r* = 0.33, *P* = 0.237; piperacillin-tazobactam: *r* = 0.39, *P* = 0.149). Actual PBP substitutions and MICs are shown in Fig. 3A. The greatest increases in cephalosporin MICs were associated with the highest numbers of substitutions; for example, the cefuroxime MIC increased from 376 to 1,506 μg/mL in ST3(001) (four substitutions) and ST17(018) (five substitutions). The cephradine MIC increased from 36 to 239 μg/mL in the latter. Intriguingly, the wild-type ancestors of these four PBP-substituted lineages had cefotaxime MICs which were still higher than those of the four lineages which have not yielded PBP-substituted strains: cefuroxime, 128 μg/mL versus 376 μg/mL, and cefotaxime, 128 μg/mL versus 256 μg/mL.

### Phylogenetic analyses.

**FIG 4:**
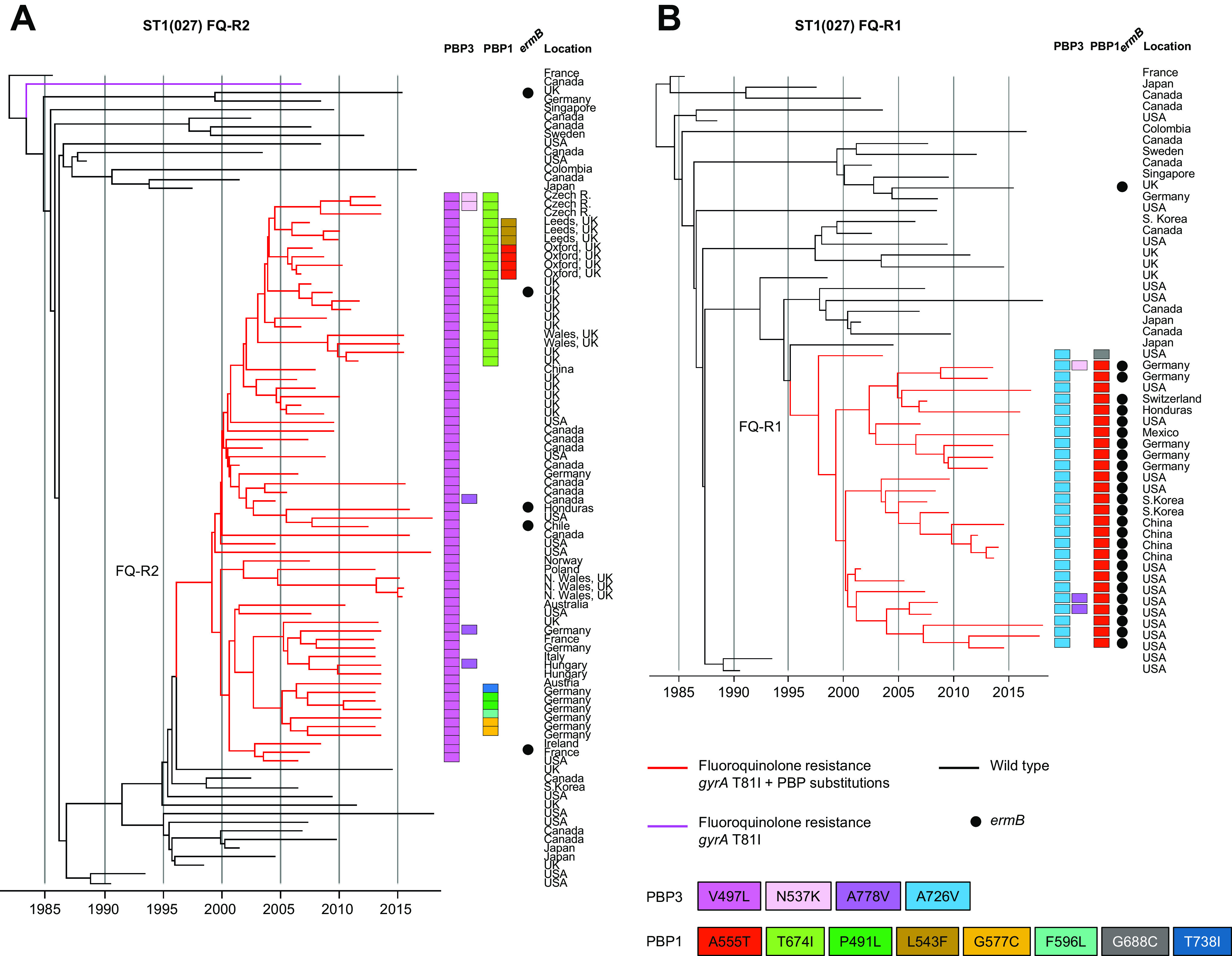
Phylogenetic analysis of lineage ST1(027). (A) Dated phylogeny to show the emergence of lineage ST1(027) FQ-R1 (*n* = 27) (19) (red branches) from the wild-type (*n* = 27). (B) Dated phylogeny to show the emergence of lineage ST1(027) FQ-R2 (*n* = 67) (19) (red branches) from the wild-type (*n* = 27). Genomes were chosen to maximize the temporal and geographic spread of wild-type and AMR strains and to represent the diversity of PBP substitutions detected. AMR determinants and PBP substitutions are as indicated in the key. Co-occurrence of fluoroquinolone resistance and PBP substitutions is highlighted by red branches. PBP substitutions are highlighted by colored squares.

**FIG 5:**
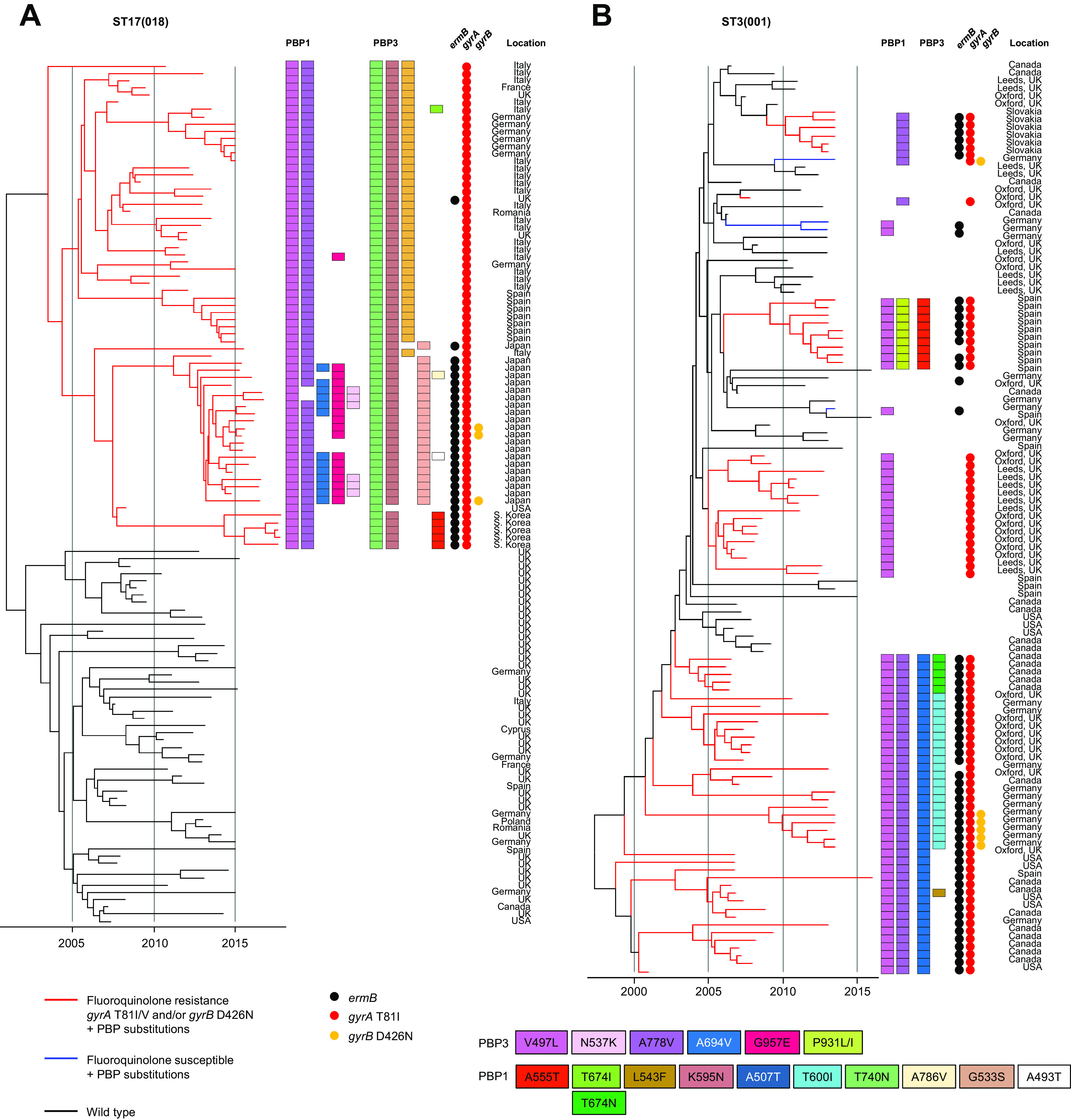
Phylogenetic analysis of lineages ST17(018) and ST3(001). (A) Dated phylogeny to show the emergence of MDR lineage ST17(018) (*n* = 66) (red branches) from the wild-type (*n* = 53). MDR strains from Europe, Southeast Asia, and North America were chosen to maximize the geographic spread and PBP substitutions. These and other AMR determinants and PBP substitutions are as indicated in the key. Co-occurrence of fluoroquinolone resistance and PBP substitutions is highlighted by red branches. (B) Dated phylogeny showing the evolutionary relationship of wild-type (*n* = 40) and PBP-substituted ST3(001) genomes (*n* = 77).

**FIG 6:**
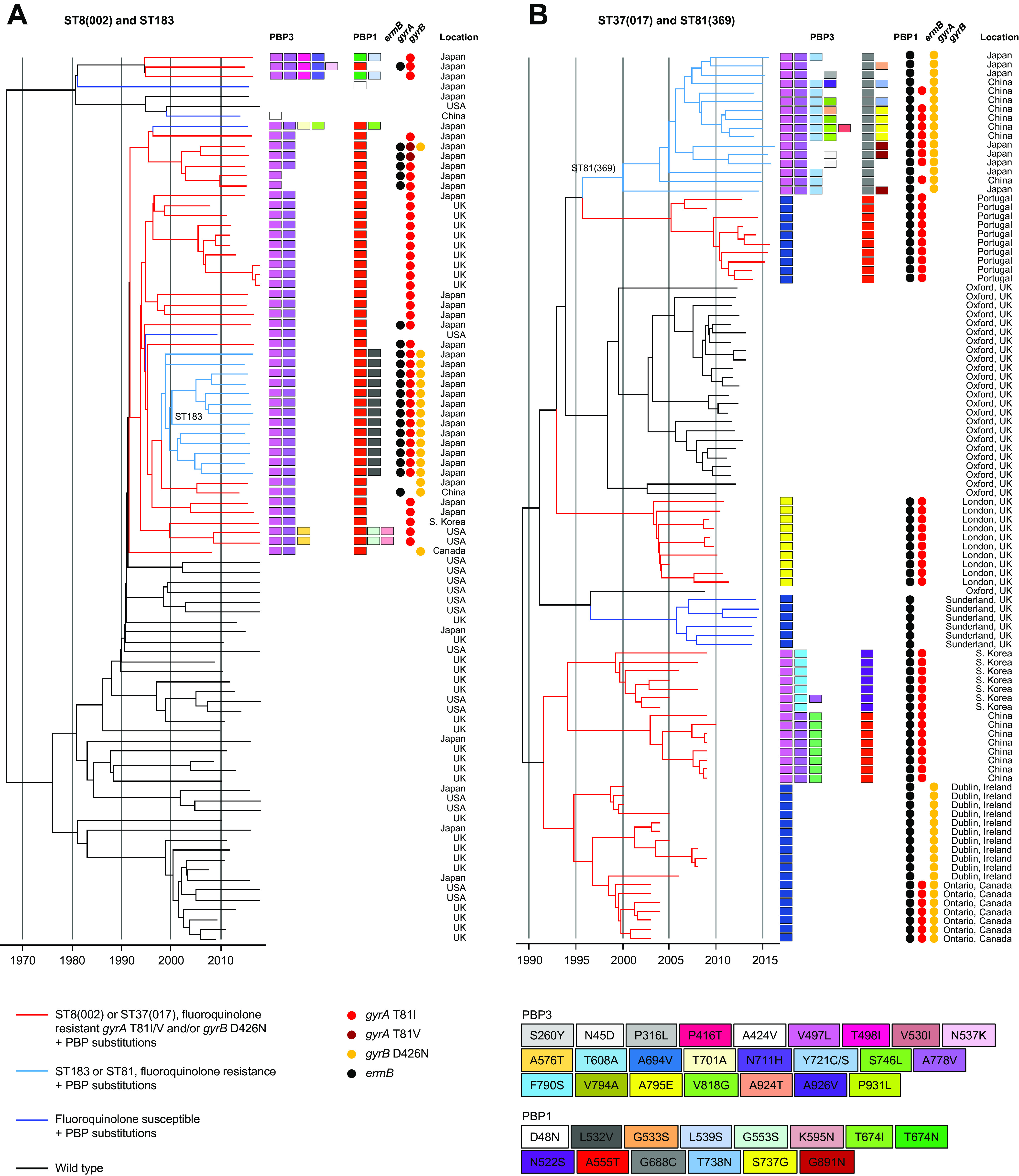
Phylogenetic analysis of lineages ST37(017)/ST81(369) and ST8(002)/ST183. (A) Dated phylogeny showing the evolutionary relationship of wild-type (*n* = 43) and PBP-substituted ST8(002)/ST183 strains (*n* = 47). Wild-type genomes were chosen to maximize genetic diversity (inferred using a previously constructed phylogeny [30]). The MDR ST183 clade was identified here as emergent from MDR ST8(002). Co-occurrence of fluoroquinolone resistance and PBP substitutions is indicated by red branches in ST8(002) and light blue branches in ST183. (B) Dated phylogeny showing the evolutionary relationship of wild-type (*n* = 25) and PBP-substituted ST37(017) (*n* = 59) and ST81(369) (*n* = 16) genomes. The MDR ST37(017) genomes were chosen to include representatives of strains associated with well documented outbreaks and other potential clusters identified by location and PBP substitution patterns. The MDR ST81(369) clade was identified here as emergent from MDR ST37(017). The majority of available wild-type ST37(017) genomes represented health care-associated and asymptomatically carried (infant) strains from a single location (Oxfordshire, United Kingdom) (30, 71). Co-occurrence of fluoroquinolone resistance and PBP substitutions is indicated by red branches in ST37(017) and light blue branches in ST81.

**FIG 7:**
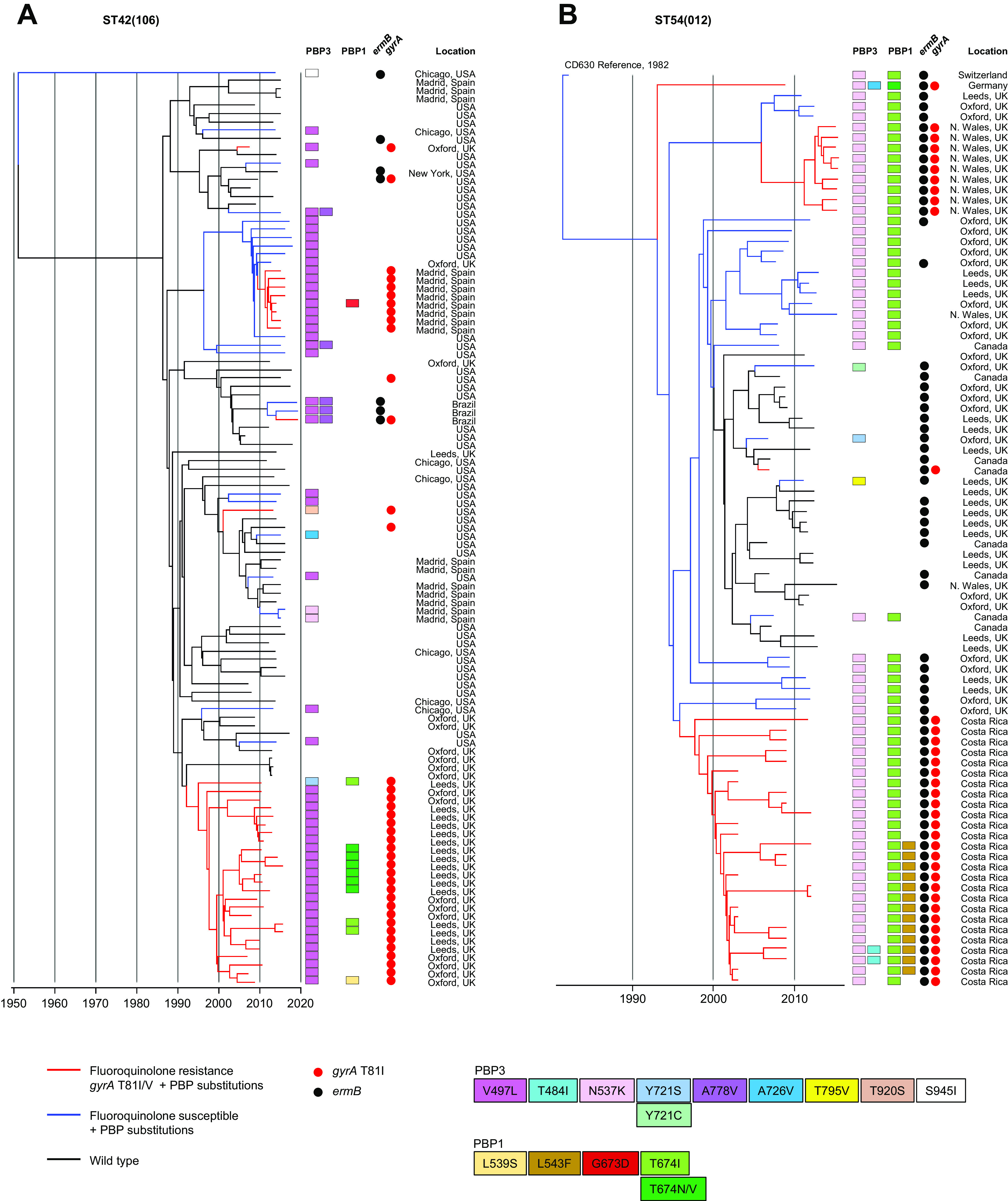
Phylogenetic analysis of lineages ST42(106) and ST54(012). (A) Dated phylogeny for the ST42(106) lineage (*n* = 111 genomes) showing the evolutionary relationship of wild-type and fluoroquinolone-resistant and/or PBP-substituted strains, chosen to represent overall diversity in terms of locations and PBP substitution patterns. (B) Same as described for panel A but for the ST54(012) lineage (*n* = 107 genomes). In both panels A and B, the co-occurrence of fluoroquinolone resistance and PBP substitutions is indicated by red branches and PBP substitutions alone are indicated by blue branches.

Recombination-corrected phylogenies were constructed to identify and date the order in which PBP substitutions and fluoroquinolone resistance were acquired by seven genetic lineages. These included the four PBP-substituted lineages which had been phenotyped [ST1(027), ST17(018), ST3(001), and ST42(106)] and a further three lineages containing notable MDR PBP-substituted fluoroquinolone-resistant strains. These were ST8(002)/ST183 and ST37(017)/ST81, both important in Southeast Asia, and ST54(012), which is notable in Costa Rica (47–51) (Fig. 4 to 7).

Irrespective of lineage, the sequence of PBP substitution acquisitions in epidemic strains typically started with PBP3 V497L and/or A778V (i.e., the most frequent PBP3 substitutions) (Table 2). Then, further PBP substitutions followed, yielding a variety of patterns. Among lineages well known for epidemic spread, the initial PBP3 V497L substitution occurred simultaneously with fluoroquinolone resistance (Fig. 4 to 7). One notable exception was the ST1(027) FQ-R1 lineage in which the PBP3 A726V substitution occurred first, while PBP3 V497L was absent (Fig. 4B).

**FIG 8:**
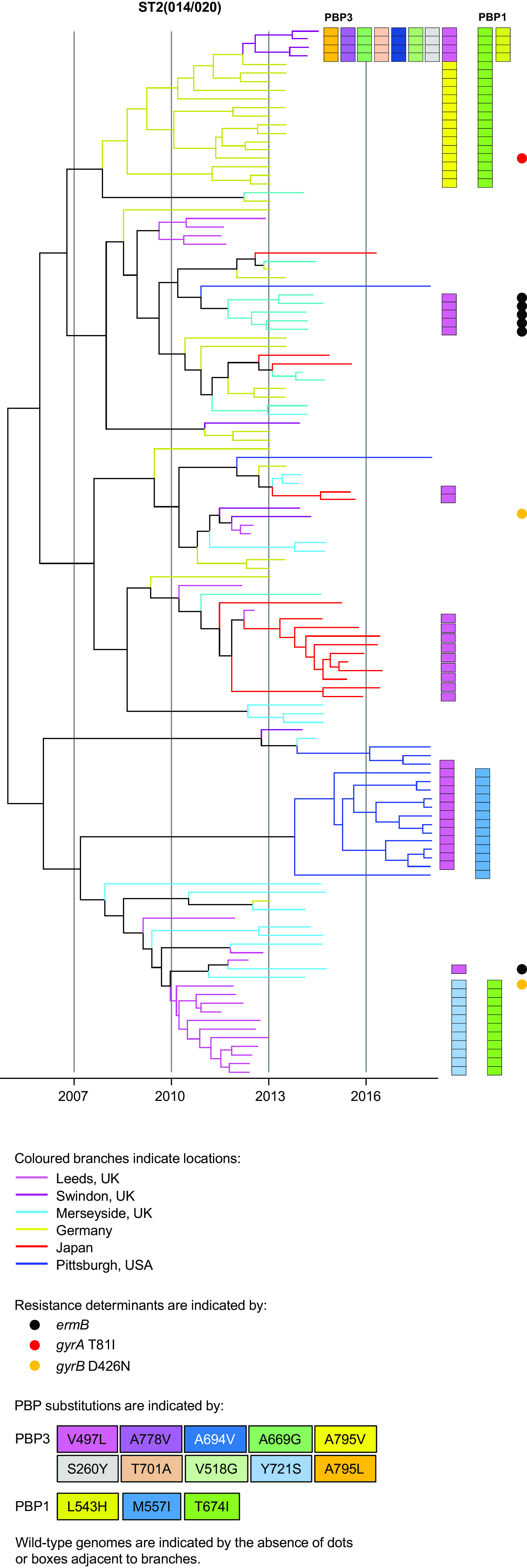
Phylogenetic analysis of ST2(014/020) genomes. Dated phylogeny showing the evolutionary relationship between wild-type (*n* = 63) and PBP-substituted ST2(014/020) strains (*n* = 60) mostly lacking fluoroquinolone resistance. Branch color indicates one of six locations indicated in the key. Clustering of PBP-substituted genomes is compared with that of the wild-type for six independent geographic locations. Wild-type genomes from each location were collected concurrently with the PBP-substituted strains. These are indicated by the absence of AMR determinants (boxes or dots). Occurrence of AMR determinants is as defined in the key.

PBP-substituted, fluoroquinolone-resistant clades evolved more than once in ST1(027), ST3(001), ST37(017)/ST81(369), ST42(106), and ST54(012) (Fig. 4A and B, 5B, 6B, and 7A and B). Their PBP substitution patterns each showed geographic structure (Fig. 4 to 8). MDR clades with the highest numbers of PBP substitutions were identified within ST17(018) in Italy and Southeast Asia (Fig. 5A), ST3(001) in United Kingdom/Germany (Fig. 5B), ST8(002)/ST183 in Japan (Fig. 6A), and ST37(017)/ST81(369) in Southeast Asia (Fig. 6B).

The occurrence of PBP substitutions in the absence of fluoroquinolone resistance was investigated phylogenetically in ST2(014/020) (Fig. 8) and to a lesser extent in ST42(106) and ST54(012) (Fig. 7). For ST2(014/020), a dated phylogeny was constructed using PBP-substituted genomes from six independent locations together with wild-type genomes from the same locations and dates (Fig. 8). The PBP-substituted genomes clustered by location, while the wild-type strains did not. It is not known whether the clusters represented within-hospital nosocomial outbreaks; their small scale suggests that at the time they occurred, such outbreaks may have been difficult to distinguish from background sporadic cases. Interestingly, both ST42(106) and ST54(012) phylogenies (Fig. 7) contained clades in which PBP substitution acquisition preceded fluoroquinolone resistance. ST42(106) recently replaced ST1(027) as the most prevalent lineage in North America (8), but a single MDR clade was not apparent, with PBP3 V497L occurring on multiple independent occasions within the ST42(106) phylogeny.

### Evolutionary mechanisms of PBP substitution acquisition.

The almost total absence of nonsynonymous SNPs within each lineage suggested that the predominant mechanism underlying the accumulation of amino acid substitutions in PBP3 and PBP1 is the stepwise fixation of *de novo* point mutations rather than the import of novel variants by horizontal genetic exchange. However, two important recombination events were identified by the co-import of synonymous and nonsynonymous SNPs, which changed PBP variants within toxin A^−^B^+^ ST81(369) (Fig. S1) and in ST1(181) relative to their ancestral A^−^B^+^ ST37(017) wild-type and ST1(027) wild-type, respectively. The ST81(369) PBP3 gene was acquired as part of a very long (~150-kb) recombination event, the donor being closely related to ST8(002) (Fig. S1). In ST1(181), described in Greece and Romania (52, 53), recombination events affected both PBP1 and PBP3, but their ~400-kb separation around the chromosome suggests that the events were independent: (i) the PBP3 allele 100 was identical to that of clade 1 ST17(018), indicating interclade 1/2 recombination, and (ii) PBP1 allele 360 contained 14 SNPs and 6 amino acid differences relative to wild-type ST1.

10.1128/mbio.00243-23.1 FIG S1 ST81(369) PBP3 was acquired via a long recombination event. Distance plots show pairwise comparisons of the donor ST8(002), recipient ST37(017), and recombinant ST81(369), with the aim of identifying the size and location of the recombination event. The genomes used were ST8(002) isolate W0003a (NZ_CP025047.1) (Yin et al., 2018), ST37(017) isolate M68 (NC_017175.1) (He et al., 2010), and ST81(369) isolate 28 WGS:QNWI01 (BioProject no. PRJNA479396, Assembly accession no. GCA_003326885.1) (Wu et al., 2019). The plots extend from 0.9 to 1.4 Mbp relative to M68 (*x* axis) so the locations of both PBP1 and PBP3 are covered: PBP1 at positions 907056 to 904363 (gene CDM68_RS04280) and PBP3 at positions 1219021 to 1221999 (gene CDM68_RS05670), indicated by vertical red dashed lines. Missing lines indicate alignment gaps. The large recombination event involves ~150 kbp (~3.5% of the 4,308,325-bp genome). A much smaller region occurred near the middle of the large event (position ~1.22 Mbp), where ST81(369) no longer resembles ST8(002) but resembles ST37(017) instead. It appears likely that this short region, adjacent to PBP3, has recombined back with ST37(017). Download FIG S1, PDF file, 0.6 MB. Copyright © 2023 Dingle et al. 2023 Dingle et al. https://creativecommons.org/licenses/by/4.0/ This content is distributed under the terms of the Creative Commons Attribution 4.0 International license.

## DISCUSSION

To date, studies aiming to determine β-lactam resistance mechanisms in C. difficile have focused on the endogenous C. difficile class D β-lactamase (37, 38). PBP substitutions have been reported only occasionally, associated with raised carbapenem MICs in a single lineage (45, 54). PBPs with reduced β-lactam affinity are clinically important in other Gram-positive pathogens, for example, Streptococcus pneumoniae (43) and methicillin-resistant Staphylococcus aureus (55). The present study was therefore performed to investigate systematically, and phenotypically, recent PBP substitutions among clinically important C. difficile lineages.

We identified multiple, recent PBP substitutions, which are focused in the conserved functional domains of the two HMW C. difficile transpeptidases, PBP1 and PBP3 (Table 2; Fig. 1). The locations of the substitutions suggest a possible response to β-lactam selective pressure, leading to changes which could (i) reduce β-lactam access to the transpeptidase active site and (ii) represent compensatory changes preserving the efficiency of cell wall biosynthesis. Further insights may be gained if the structures of these proteins are determined. Equivalent changes were not detected in LMW PBP2 and PBP4. This difference between the two PBP classes may reflect differences in function (46); for example, HMW PBP1 and PBP3 are essential for growth *in vitro*, while LMW PBP2 and PBP4 are not essential for growth but are required for sporulation (46). Although PBP5 transpeptidase was variably present, it was not recently acquired by MDR C. difficile lineages. Its constant chromosomal location suggests that gradual loss rather than recent acquisition may explain its variable presence.

PBP1 and PBP3 substitutions were significantly associated with raised cephalosporin MICs, relative to closely related wild-type strains, and the higher the number of substitutions, the higher the MIC (Fig. 3A). The mechanism underlying substitution acquisition was not recombination but rather the accumulation of *de novo* chromosomal mutations. This was indicated because virtually all SNPs were nonsynonymous, flanked the catalytic domains (Fig. 1), and arose multiple times (Table 2). Only two major recombination events were found involving ST81(369) (see Fig. S1 in the supplemental material) and ST1(181). While we have shown statistically significant associations between PBP substitutions and raised beta-lactam MICs in isolates separated from the wild-type by small numbers of SNPs (Fig. 3A), the demonstration of a causal association would require controlled laboratory allelic exchange experiments, which are beyond the scope of the present study.

The Co-occurrence of PBP substitutions and fluoroquinolone resistance in the clinically important epidemic lineages (Fig. 2) was statistically significant. Stewardship of cephalosporins may represent an additional tool for outbreak control, potentially mirroring the success of fluoroquinolone stewardship (30). In support of this, studies performed between the introduction of first-generation cephalosporins (mid-1960s) and the emergence of widespread fluoroquinolone resistance (late 1980s onwards) reported cephalosporin stewardship alone to be successful (24, 56–58). We found here that the cephalosporin MICs of wild-type strains differed according to whether they later went on to acquire PBP substitutions (Fig. 3A). For example, wild type cefuroxime and cefotaxime MICs were 128 μg/mL when no PBP substitutions subsequently acquired, versus wild type MICs of 376 μg/mL or 256 μg/mL when PBP substitutions were subsequently acquired. The mechanism underlying these differences must predate PBP substitution acquisition and is currently unknown. It suggests that wild-type precursors of PBP-substituted clades are better adapted to cephalosporin exposure and therefore perhaps more likely to acquire PBP substitutions, as observed for the multiple clades detected phylogenetically in specific lineages. The co-occurrence of PBP substitutions and fluoroquinolone resistance currently hinders attempts to understand their relative importance in epidemic spread. Further controlled studies of cephalosporin and fluoroquinolone stewardship are needed.

Given the large number of PBP-substituted, clinically important MDR clades which have emerged over the last 30 years in different geographic regions (Fig. 4 to 8), it is surprising that their elevated cephalosporin MICs have not been highlighted previously. This likely reflects acceptance of the concept that high cephalosporin MICs (~256 μg/mL) are inherent to C. difficile (10, 33–36) and that their determination is consequently uninformative, particularly since wild-type MICs are thought to exceed clinically relevant concentrations and a mechanism linked to raised MICs has not been defined.

In addition to PBP substitutions, our phylogenies revealed the sequence and timing of MDR acquisition by clinically important lineages. The co-occurrence of PBP substitutions and fluoroquinolone resistance predated epidemic spread, which was reflected in short-branched, geographically structured clades (Fig. 4 to 7). The first PBP substitution was typically PBP3 V497L, followed by others, yielding a variety of final combinations. The more highly PBP-substituted, fluoroquinolone-resistant clades were frequently positive for additional AMR determinants, particularly the *ermB* gene (clindamycin resistance) and *rpoB* substitutions (rifampin resistance) (Data Set S1; Fig. 5 and 6).

We dated the emergence of the two MDR ST1(027) clades (FQ-R1 and FQ-R2) to the mid- to late 1990s, as previously described (19) (Fig. 4A and B). The emergence date of the MDR ST17(018) clade is compatible with the first reports of outbreaks in 1996 to 1999 (59), the phylogeny root having a 95% credible interval dating of December 1998 to July 2002 (Fig. 5A). European and Asian ST17(018) clades then diverged, acquiring further region-specific PBP substitutions (Fig. 5A), arguing against their recent intercontinental spread.

Greater numbers of PBP substitutions were significantly associated with the highest cephalosporin MICs (Fig. 3A). Consistent with this, highly substituted clades of multiple lineages [ST17(018), ST81(369)/ST37(018), and ST183/ST8(002)] (Fig. 5A and 6A and B) predominated in Southeast Asia, where cephalosporin use is high (60–62). Adaptation to local prescribing conditions through PBP substitution acquisition offers a possible explanation for the temporal and geographic variation in prevalent C. difficile lineages (6, 8, 19, 47, 58, 63). For example, United Kingdom and local (Oxfordshire) cephalosporin prescribing levels from 1998 to 2013 were described previously (30) and were higher (as was fluoroquinolone prescribing) when epidemic lineages (ST1(027), ST3(001), and ST42(106)) predominated. This concept may extend to competitive exclusion of lesser PBP-substituted strains by more highly substituted ones, a scenario requiring greater numbers of PBP substitutions to carry a fitness cost. This appears possible, as in Clostridium perfringens *in vitro*, where PBP substitutions are associated with slower growth (64). We hypothesize that local levels of cephalosporin (and fluoroquinolone) prescribing determine the prevalent C. difficile MDR strain(s) in a given region. For example, the unusually low levels of ST1(027) seen in Asia (65) may reflect competitive exclusion, under local prescribing conditions, by the more highly PBP-substituted clades which predominate here. The relative geographic restriction of ST1(027) FQ-R1 (in the United States, South Korea, and Germany) in comparison to the more globally distributed FQ-R2 may also reflect the different PBP substitutions of the two clades (Fig. 4) and variations in MIC (Fig. 3A).

MDR strains exhibit high transmissibility in clinical settings when prescribing is uncontrolled (17). The PBP1 and PBP3 transpeptidases function in cell wall biosynthesis, and therefore, substitutions impacting their catalytic domain could affect transmissibility via sporulation. A high sporulation phenotype has been reported in at least two epidemic lineages: ST3(001) (United Kingdom) and ST81(369) (Asia) (66, 67). Sporulation phenotype is reportedly variable in ST1(027) (68, 69), and we have observed variation in its PBP substitutions (Fig. 4A and B; Data Set S1). However, the possibility of a link remains to be investigated. It is relevant to future experimental design that the MDR laboratory reference strains CD630 [ST54(012)] and R20291 [ST1(027)] both contain PBP substitutions (Table 1).

PBP substitutions occurred without fluoroquinolone resistance in a minority of the toxigenic lineages studied: ST42(106), ST37(017), ST54(012), ST2(014/020), and ST6(005) (Fig. 2; Data Set S1). ST42(106) was of interest since it recently exceeded the prevalence of ST1(027) in North America (8, 70). Visual inspection of phylogenies was used to assess whether PBP substitutions might enhance transmissibility in the absence of fluoroquinolone resistance. An ST2(020/014) phylogeny showed some possibility of locally enhanced transmission (Fig. 8), as did ST54(012) and ST42(106) (U.S. genomes) (Fig. 7A and B). However, these events were small scale, and this question remains to be answered. PBP substitutions (without fluoroquinolone resistance) were, however, widespread within the two nontoxigenic lineages ST15(010) and ST26(039) (Table S1), potentially explaining their high prevalence over other nontoxigenic strains. As well as being isolated coincidentally from patients with CDI caused by toxigenic strains, they are frequently carried by human infants (71) and potentially pets and farm/wild animals. The niches they colonize are likely to be more extensive than, and distinct from, the MDR toxigenic lineages, leading to potential opportunities for β-lactam exposure in the absence of exposure to fluoroquinolones, for example, in community or veterinary settings or in the environment. In contrast, the MDR toxigenic lineages containing both PBP substitutions and fluoroquinolone resistance-associated SNPs exist in a much narrower ecological niche, persisting only in health care settings when prescribing of β-lactams and/or fluoroquinolones is uncontrolled (30). Examples of isolates containing PBP substitutions but lacking other AMR determinants were not included among those we phenotyped. This limitation is mitigated to some extent by the fact that such genomes do have specific PBP substitutions in common with the phenotyped strains, such as PBP3 V497L, but would be interesting to examine in the future.

The cephalosporin MICs for PBP-substituted strains were extremely high for certain antibiotics (for example, >512 μg/mL for cefotaxime and up to 1,506 μg/mL for cefuroxime) (Fig. 3A). This raises questions about the *in vivo* conditions required for PBP substitution selection. Intravenous β-lactams are eliminated in active form by biliary excretion, resulting in highly variable intestinal concentrations (72). Intestinal concentrations ranging from 1.01 to 1,345 μg/mL have been reported (73), and so the potential exists for C. difficile to be exposed *in vivo* to cephalosporin concentrations reaching the MICs measured here. Bacteria with raised MICs can also be selected experimentally at antimicrobial concentrations up to several hundred-fold below lethal levels. However, the overall contribution made by such “sub-MIC selection” to resistance in clinically important bacteria is unknown (74–79).

To date, <1% of known C. difficile lineages (1,042 STs identified as at 20 January 2023; https://pubmlst.org/organisms/clostridioides-difficile/) have evolved a PBP-substituted clade(s). Furthermore, each lineage has tended to evolve more than one such clade (Fig. 4 to 7). This suggests that the wild-type phenotype of such lineages may favor the acquisition of chromosomal SNPs which are associated with raised cephalosporin MICs. As discussed above, the wild-type cephalosporin MICs for PBP-substituted lineages were indeed higher than for those lacking such strains (Fig. 3A). This potentially favors survival of these lineages in low cephalosporin concentrations *in vivo*, which could allow selection of PBP substitutions to occur. Since such strains also frequently contain additional SNPs associated with MDR, an alternative or additional mechanism might be a hypermutator phenotype, as in S. pneumoniae (74). An interesting area for further work could be a genome-wide association study to detect SNPs associated with raised cephalosporin MICs, an approach already employed to identify three novel mutations associated with reduced susceptibility to metronidazole (80).

In summary, our findings identify a potential role for cephalosporin selection in the evolution of epidemic CDI lineages. Specific regional prescribing practices may determine the locally predominant epidemic strains, potentially explaining the marked international variation in C. difficile molecular epidemiology. Since antimicrobial stewardship typically targets multiple drug classes (29, 81) and epidemic strains have raised MICs for fluoroquinolones, cephalosporins (Fig. 2 and 3), and more variably clindamycin (*ermB*) (Data Set S1), it is difficult to determine the relative contributions made by stewardship of each drug to CDI control. The timing of cephalosporin and fluoroquinolone resistance acquisition, immediately before the emergence of multiple epidemic strains from divergent C. difficile genetic backgrounds, suggests that AMR may be equally important as, or even exceed, strain-specific virulence determinants in driving epidemic CDI.

## MATERIALS AND METHODS

Whole-genome sequencing (WGS) from 7,094 C. difficile isolates, predominantly cultured from humans with CDI, was obtained. Clinical isolates from hospital and community patients from Europe, North and South America, Southeast Asia, and Australia were included. Fourteen CDI lineages and two nontoxigenic lineages were represented. A complete list of genomes, their identifiers in public databases, and references are provided (see Data Set S1 in the supplemental material). Raw sequence reads were assembled *de novo* as required using Velvet (version 1.0.7 to 1.0.18) (82) and VelvetOptimiser with default settings (2.1.7) (83). A minority of genomes were obtained assembled, either from the NCBI database (https://www.ncbi.nlm.nih.gov/genome/browse#!/prokaryotes/535/) or EnteroBase (https://enterobase.warwick.ac.uk/species/index/clostridium) (84). Assemblies were imported to a BIGSdb database (85) which was used to identify the seven loci used in multilocus sequence typing (44). Sequence types (STs) were assigned using the C. difficile PubMLST database (https://pubmlst.org/organisms/clostridioides-difficile/). STs and PCR ribotypes were used to indicate genetic lineages, identified by, e.g., the notation ST1(027) [sequence type 1(PCR ribotype 027)].

BLAST searches performed within BIGSdb (85) were used to identify and extract chromosomal gene sequences for PBP transpeptidases (PBP1–5) (45), together with *gyrA*, *gyrB*, and *rpoB*, specific mutations in which confer AMR. Established amino acid substitutions scored as conferring resistance to fluoroquinolones were GyrA T81I and GyrB D426N, and those scored as conferring resistance to rifampin were RpoB R505K, H502N, and S498T. Acquisition of *ermB*, conferring clindamycin resistance, was also noted (86–89). Each unique allele sequence identified at these loci (PBP1–5, *gyrA*, *gyrB*, *rpoB*, and *ermB*) was assigned a number (Data Set S1) and can be downloaded at https://pubmlst.org/organisms/clostridioides-difficile/ (44, 85). Newly extracted gene sequences were queried against this database, and the allele numbers were recorded for each genome together with the substitutions relevant to AMR (Data Set S1).

### Identification of recent PBP substitutions.

Identification of recent PBP substitutions was achieved using MEGA (https://www.megasoftware.net/) (90), which facilitated within-lineage comparisons of the nucleotide and amino acid sequences of PBP1–5 alleles. Comparisons were made relative to the wild-type PBP sequence for each lineage, wild-type alleles being taken from non-MDR genomes within the lineage.

### Phenotyping.

Isolates were chosen for phenotyping from a total of eight lineages, four containing both PBP-substituted and wild-type strains and four containing wild-type strains only. To control for any potential confounding due to population structure, the isolates containing PBP substitutions were chosen to minimize SNP distances from their equivalent wild-type (Fig. 3A). The following numbers of isolates were phenotyped per lineage: ST1(027) FQ-R1 and FQ-R2, total phenotyped = 7, comprising 2 wild-type and 5 PBP substituted; ST3(001), total phenotyped = 4, comprising 1 wild-type and 3 PBP substituted; ST17(017), total phenotyped = 2, comprising 1 wild-type and 1 PBP substituted; and ST42(106), total phenotyped = 2, comprising 1 wild-type and 1 PBP substituted. Among the wild-type-only lineages, the following were phenotyped: ST10(015) (*n* =11), ST6(005) (*n* = 10), ST56(058) (*n* = 4), and ST7(026) (*n* = 5).

MICs of cefotaxime, cefuroxime, cephradine, amoxicillin, amoxicillin-clavulanate, meropenem, imipenem, and piperacillin-tazobactam were determined by Wilkins-Chalgren agar dilution methods (91, 92). Briefly, C. difficile isolates and controls (C. difficile ATCC 700057, C. difficile E4 [PCR ribotype 010] and Bacteroides fragilis ATCC 25285) were cultured in prereduced Schaedler anaerobic broths at 37°C for 24 h anaerobically. Isolates and controls were diluted in prereduced saline to a McFarland standard 1 equivalence and multipoint inoculated onto prepared antibiotic-containing agar plates and controls. Agar plates were incubated at 37°C for 24 h, anaerobically, prior to MIC determination. The MIC was defined as the lowest concentration of antimicrobial that completely inhibited growth, showed a marked reduction in growth, showed only 1 or 2 colonies, or left a faint haze of growth on the plate, according to Clinical and Laboratory Standards Institute (CLSI) guidelines (93).

Antimicrobial concentrations were prepared using solvents and diluents recommended in the CLSI guidelines (93, 94). For amoxicillin-clavulanate and piperacillin-tazobactam, clavulanic acid and tazobactam were added to agar at fixed concentrations of 2 mg/L and 4 mg/L, respectively. In order to test susceptibility within normal doubling dilutions, additional antibiotic concentrations were prepared for the antibiotic plate range. All antibiotics were tested at the following dilutions: 0.125, 0.25, 0.5, 1, 2, 4, 8, 16, 32, 36, 40, 46, 53, 64, 71, 80, 91, 107, 128, 142, 160, 182, 213, 256, 284, 320, 366, 427, and 512 mg/L. Additionally, cefuroxime and cephradine were prepared up to their limit of solubility, and the following ranges of dilutions were prepared: for cefuroxime, 102, 120, 143, 160, 205, 213, 240, 287, 319, 409, 478, and 572 mg/L; for cephradine, 105, 118, 134, 157, 188, 209, 235, 269, 314, 376, 418, 471, 538, 627, 753, 837, 941, 1,076, 1,255, and 1,506 mg/L.

### Statistical methods.

The association between the presence of PBP substitutions and the presence of fluoroquinolone resistance substitutions was tested using a two-tailed Fisher’s exact test. This was done for each lineage containing at least one isolate with PBP and/or fluoroquinolone resistance substitutions. Lineages were further combined into three groups: (i) clinically important, toxigenic lineages with evidence of multidrug resistance, (ii) other toxigenic lineages lacking MDR, and (iii) nontoxigenic lineages. The correlation between the number of PBP substitutions and β-lactam MICs was calculated for each antibiotic, using Spearman’s rank correlation coefficient. This was done for all isolates in lineages containing PBP substitutions [ST1(027), ST17(018), ST3(001), and ST42(106)].

### Construction of dated phylogenies.

Dated phylogenies were constructed using genomes chosen to maximize the geographic and temporal spread of wild-type and AMR strains, as well as to represent the diversity of PBP substitutions detected in each lineage. Each set of genomes was first aligned to a reference using MuMMER version 3.1 (95) to produce a genome-wide alignment. The following genomes were used as references; ST1(027) strain R20291, accession no. FN545816 (96) (Fig. 4); ST17(018) strain CBA7209, accession no. QLOB00000000; (97) (Fig. 5A); ST3(001) strain BI9, accession no. FN668944.1 (96) (Fig. 5B); ST8(002) and descendant ST183 strain W0003a, accession no. CP025047.1 (98) (Fig. 6A); ST37(017) and descendant ST81(369) strain M68, accession no. NC_017175.1 (96) (Fig. 6B); ST42(106) strain W0023a, accession no. CP025045.1, (98) (Fig. 7A); ST54(012) strain CD630, accession no. AM180355.1 (99) (Fig. 7B); and ST2(014/020) strain W0022a, accession no. CP025046.1 (98) (Fig. 8).

Initial phylogenies, built using PhyML version 3.3 (100), were then corrected for recombination using ClonalFrameML version 1.12 (101). Finally, these phylogenies were dated using BactDating version 1.1 (102) by assuming a mean evolutionary rate of 1.4 mutations per year per genome as in previous similar studies (103).
