# Supplementary material for: Penicillin Binding Protein Substitutions Cooccur with Fluoroquinolone Resistance in Epidemic Lineages of Multidrug-Resistant Clostridioides difficile
Source: mBio. 2023 Apr 5;14(2):e00243-23. doi: 10.1128/mbio.00243-23 (PMC10128037; doi:10.1128/mbio.00243-23)
Supplement: TABLE S1 [file mbio.00243-23-s0003.docx]

**Table S1**. **PBP1 C-terminal Repeat Sequences, exemplified by the PBP1 alleles shown.**

| PBP1 Allele* | Translated length (aa) | C-terminal repeats (n) | Top 3 Blastp hits with 100% alignment length coverage  (NCBI Accession no.) | Blastp  e value | Identity aa  (%) | Alignment Length (aa) | C-terminal repeats within alignment (n) |
| --- | --- | --- | --- | --- | --- | --- | --- |
| 344 | 925 | 6 | WP_236733009.1 | 0.0 | 99.46 | 925 | 6 |
|  |  |  | WP_236733684.1 | 0.0 | 99.35 | 925 | 6 |
|  |  |  | WP_236733556.1 | 0.0 | 99.24 | 925 | 6 |
|  |  |  |  |  |  |  |  |
| 12 | 897 | 4 | HBE8744475.1 | 0.0 | 100 | 897 | 4 |
|  |  |  | HBH3588122.1 | 0.0 | 99.89 | 897 | 4 |
|  |  |  | WP_042520389.1 | 0.0 | 99.89 | 897 | 4 |
|  |  |  |  |  |  |  |  |
| 11 | 883 | 3 | WP_109285206.1 | 0.0 | 100 | 883 | 3 |
|  |  |  | WP_131780696.1 | 0.0 | 99.89 | 883 | 3 |
|  |  |  | VHS46864.1 | 0.0 | 99.77 | 883 | 3 |
|  |  |  |  |  |  |  |  |
| 8 | 869 | 2 | WP_021422853.1 | 0.0 | 100 | 869 | 2 |
|  |  |  | VHT18253.1 | 0.0 | 99.88 | 869 | 2 |
|  |  |  | WP_133134640.1 | 0.0 | 99.88 | 869 | 2 |
|  |  |  |  |  |  |  |  |
| 39 | 855 | 1 | WP_022621042.1 | 0.0 | 100 | 855 | 1 |
|  |  |  | WP_232863308.1 | 0.0 | 99.88 | 855 | 1 |
|  |  |  | WP_131008311.1 | 0.0 | 99.88 | 855 | 1 |
|  |  |  |  |  |  |  |  |

No examples containing 5 repeats were found.

*PBP1 allele sequences are available for download at <https://pubmlst.org/bigsdb?db=pubmlst_cdifficile_seqdef&page=downloadAlleles>

The variable PBP1 length was caused by variable numbers of a 42 nt sequence which occurred between one and six times, near the 3’ end of the gene. Each repeat encoded 14 amino acids, with the sequence TPPDNGGNNGGGST. The numbers of repeats varied within ST, and did not follow patterns of MDR. When blastp searches were performed using PBP1 alleles containing different numbers of repeats, (1-4 and 6), matches with 100% identity were identified for each, suggesting that this repeat phenomenon is not specific to the methods used here. The details of the top three blastp matches for each number of repeats are shown above.
