# Supplementary material for: Penicillin Binding Protein Substitutions Cooccur with Fluoroquinolone Resistance in Epidemic Lineages of Multidrug-Resistant Clostridioides difficile
Source: mBio. 2023 Apr 5;14(2):e00243-23. doi: 10.1128/mbio.00243-23 (PMC10128037; doi:10.1128/mbio.00243-23)
Supplement: FIG S1 [file mbio.00243-23-s0001.pdf]

Figure S1

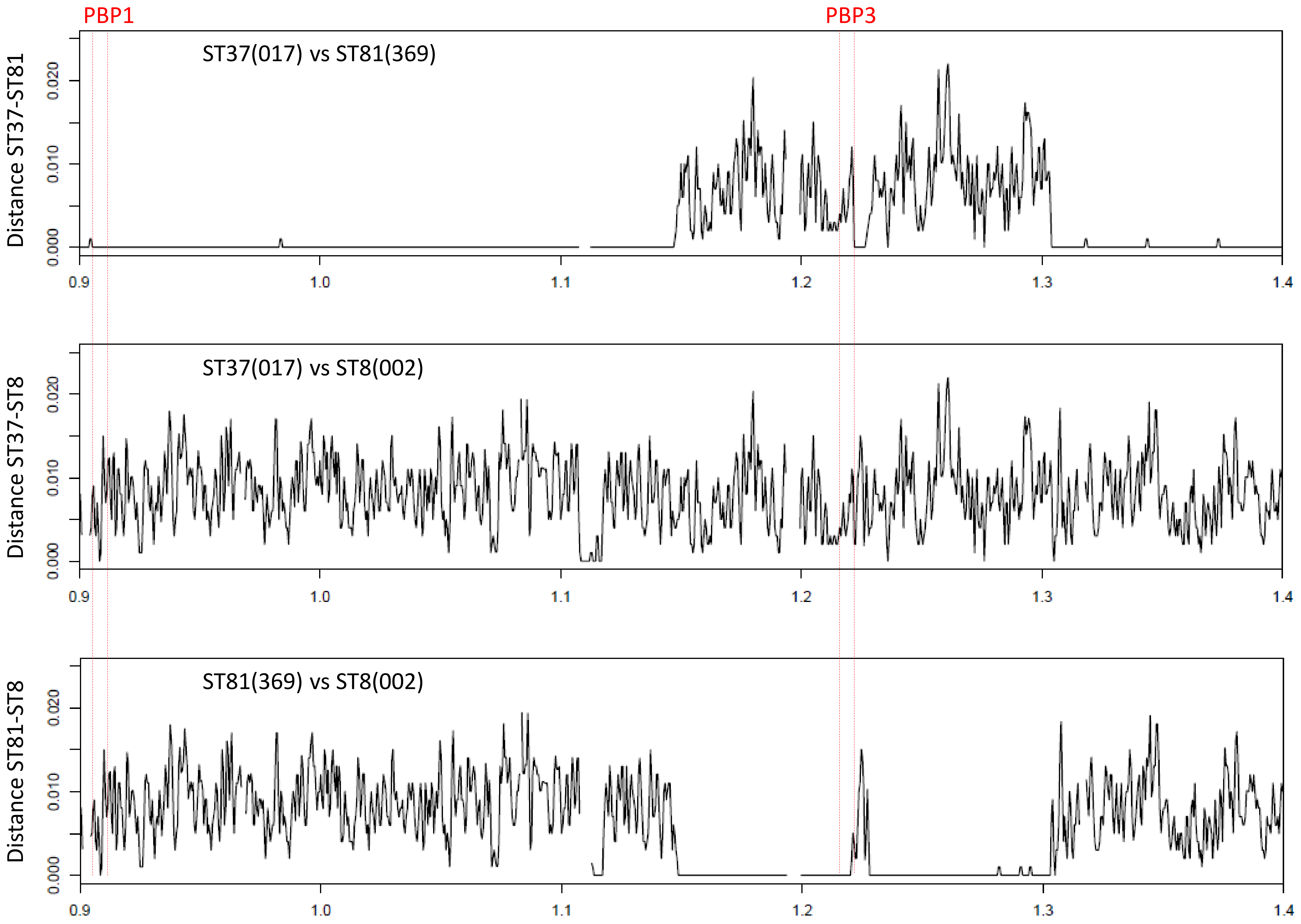

Position within *C. difficile* strain M68 genome (Mbp)

References

Yin C, Chen DS, Zhuge J, McKenna D, Sagurton J, Wang G, Huang W, Dimitrova N, Fallon JT. 2018. Complete genome sequences of four toxigenic *Clostridium difficile* clinical isolates from patients of the Lower Hudson Valley, New York, USA. Genome Announc 6(4):e01537-17.

He M, Sebaihia M, Lawley TD, Stabler RA, Dawson LF, Martin MJ, Holt KE, Seth-Smith HM, Quail MA, Rance R, Brooks K, Churcher C, Harris D, Bentley SD, Burrows C, Clark L, Corton C, Murray V, Rose G, Thurston S, van Tonder A, Walker D, Wren BW, Dougan G, Parkhill J. 2010. Evolutionary dynamics of *Clostridium difficile* over short and long time scales. Proc Natl Acad Sci USA 107:7527-7532.

Wu Y, Liu C, Li WG, Xu JL, Zhang WZ, Dai YF, Lu JX. 2019. Independent microevolution mediated by mobile genetic elements of individual *Clostridium difficile* isolates from Clade 4 revealed by whole-genome sequencing. mSystems 4(2):e00252-18.
